# A Computational Method to Quantify the Effects of Slipped Strand Mispairing on Bacterial Tetranucleotide Repeats

**DOI:** 10.1038/s41598-019-53866-z

**Published:** 2019-12-02

**Authors:** Gregory P. Harhay, Dayna M. Harhay, James L. Bono, Sarah F. Capik, Keith D. DeDonder, Michael D. Apley, Brian V. Lubbers, Bradley J. White, Robert L. Larson, Timothy P. L. Smith

**Affiliations:** 1 USDA ARS US Meat Animal Research Center, Clay Center, NE United States; 2 Texas A&M AgriLife Research, Amarillo, TX and the College of Veterinary Medicine & Biomedical Sciences, Texas A&M University System, College Station, TX United States; 3 Veterinary and Biomedical Research Center, Inc, Manhattan, KS United States; 4 Kansas State University, College of Veterinary Medicine, Manhattan, KS United States

**Keywords:** Next-generation sequencing, Genome informatics, Bacterial genomics, Bacterial pathogenesis

## Abstract

The virulence and pathogenicity of bacterial pathogens are related to their adaptability to changing environments. One process enabling adaptation is based on minor changes in genome sequence, as small as a few base pairs, within segments of genome called simple sequence repeats (SSRs) that consist of multiple copies of a short sequence (from one to several nucleotides), repeated in series. SSRs are found in eukaryotes as well as prokaryotes, and length variation in them occurs at frequencies up to a million-fold higher than bacterial point mutations through the process of slipped strand mispairing (SSM) by DNA polymerase during replication. The characterization of SSR length by standard sequencing methods is complicated by the appearance of length variation introduced during the sequencing process that obscures the lower abundance repeat number variants in a population. Here we report a computational approach to correct for sequencing process-induced artifacts, validated for tetranucleotide repeats by use of synthetic constructs of fixed, known length. We apply this method to a laboratory culture of *Histophilus somni*, prepared from a single colony, and demonstrate that the culture consists of populations of distinct sequence phase and length variants at individual tetranucleotide SSR loci.

## Introduction

Bacteria utilize several mechanisms to reversibly and rapidly switch gene expression states in response to environmental cues, including gene conversion, homologous recombination, site-specific recombination, and SSR length variation^1,2^. SSM-mediated SSR length variation can result in rapid phenotypic changes in response to environmental changes enabling adaptation. When SSR length variation affects phenotype, it is referred to as phase variation, and has been observed in multiple bacterial species^2^. For example, gram-negative mucosal pathogens including *Neisseria gonorrhoeae*, *Helicobacter pylori*, and *Haemophilus influenza* exploit SSM-mediated phase variation to adapt to host reproductive, gastric, and respiratory niches, respectively^1^. Similarly, in the normally commensal gram-positive gastrointestinal bacteria *Streptococcus gallolyticus*, SSM-mediated phase variation can cause a shift in pili expression resulting in infective endocarditis in elderly patients^3^.

The stochastic nature of SSRs make them challenging to characterize. Specifically, approaches based on locus-targeting using PCR can be complicated by artifacts during amplification, whereby the polymerase introduces changes not actually present in the genome. For example, genotyping of SSR microsatellites for genetic studies in mammalian genomes demonstrates the generation of variant amplification products (“stutter bands”), whether analyzed by gel-based sizing or by sequencing-by-synthesis (SBS) methods^4–6^. This artifact may occur even when libraries are prepared without amplification because the sequencing platform itself uses PCR for generating clusters for sequencing. In addition, both gel-based length determination and SBS-based methods have limitations with respect to the total length of SSRs that can be analyzed. In the former, the resolution of gel separation is limiting, and in the latter the limitation is the available read lengths. SBS methods were previously used in a comparative genomics approach to predict SSM-mediated SSR length variable loci involving cell surface structures or DNA metabolism (and therefore linked to virulence) in five isolates of *Neisseria meningitides*^7^. Although the library preparation avoided amplification, the SBS method performed cluster generation on the sequencing instrument by PCR methods. There was only an average of 66 read pairs reported that fully spanned the SSRs. The impact of sequence technology-induced apparent variation was assessed by examining supposedly invariant (non-phase variant) SSR within the same isolate. However, 11% of putative “non-variant” SSRs were still observed to display variable numbers of RU that could either be artifacts of the sequencing or true biological variation. A similar approach had been previously used to control for indels in C/G homopolymer tracts associated with phase variation^8^.

Long-read SMRT sequencing in circular consensus sequence (CCS) mode holds promise to improve interpretation of sequence data with respect to SSR variability, as it avoids amplification-based artifacts and corrects for sequencing error by repeatedly sequencing across both strands of the SSR to correct for errors that may occasionally occur in a single pass. Further, the CCS lengths can span essentially all SSRs present in bacteria. CCS sequencing has been previously applied to improve the characterizations of longer SSRs in mammalian genomes and identify association with disease^9–13^. However, in general, these genetic studies were designed to determine the diploid genotype of the individual, and when minor numbers of sequences were observed with lengths other than the two main genotypes, they were simply ignored. In the context of bacterial populations, it is imperative to separate experimental artifact from true genomic variation in SSR length to enable precise quantitative assessments of the impact of lowly abundant SSR length variants on the biological mechanisms affected by SSM-mediated phase variation. No previous study, to our knowledge, has performed controlled experiments designed to identify and quantify the impact of sequencing technology-based artifact on the interpretation of SSM-mediated SSR length variability in bacterial populations.

To differentiate between biological signal and sequencing-induced artifact, we created synthetic DNA duplexes of defined length mimicking the observed genomic sequences to eliminate intra-sample variability that could exist in preparations of bacterial genomic DNA. This approach supported the direct estimation of sequence artifact based on the postulate that SSM-mediated total SSR length variation must change by an integer number of SSR RUs. Where this postulate holds true, the fractional base compositions (FBC, the proportion of the number of A, C, G, or T relative to the total number of bases in the SSR) for a base in SSM-mediated SSR length variants are necessarily nearly identical and essentially independent of the total length of the SSR, given the SSR > RU length. We developed a method to identify the segment of each CCS that completely spans an SSR, defined as the region of interest (ROI), followed by extraction of the ROI from each CCS, then the application of minimum quality and maximum FBC filters. The FBC filter utilizes a z-score^14,15^, where the FBCs for each base were computed for each CCS and the dispersion of these FBC values were computed in the form of a z-score, a normalized or “standard” dimensionless form of the standard deviation. The z-score threshold is the maximum allowable absolute value of the z-scores that a given CCS must possess to pass the filter.

The SSR that we used to develop our analytical method was originally observed in a strain of *Histophilus somni*, a gram-negative mucosal inhabitant and occasional pathogen of cattle (GenBank accession CP018802 see Methods). This genome was chosen because it possessed the longest perfect tetranucleotide repeat (250 bp) that we have observed in our collection of closed bacterial genomes associated with respiratory disease. The 79 bp perfect tetranucleotide SSR (20 RU-1 bp) with RU sequence AAGC was the largest tetranucleotide SSR in this genome that we could target for chemical synthesis because there is a practical limit of 100 bp for chemically synthesized oligonucleotides that could be annealed into duplexes by commercial providers. Additionally, we wanted to maintain the X RU-1 bp phase and include sufficient flanking sequence for robust annealing which we deemed to be one turn of the DNA helix (10.5 bases), setting a minimum flank length of 11 bp. This flanking sequence was also necessary to test our methods to identify and extract CCS ROIs from extant genomic sequence, followed by removal of flanking sequence to generate the CCS ROI presented here. All the aforementioned considerations constrained the control total SSR length to be practically limited to duplexes containing 18 RU-1 bp (71 bp SSR, 96 bp oligonucleotide), 17 RU-1 bp (67 bp SSR, 92 bp oligonucleotide), or 16 RU-1 bp (63 bp SSR, 88 bp oligonucleotide) plus genome flanking sequence of 14 bp on the 5′-end of the SSR and 11 bp on the 3′-end. The only primary sequence difference between the synthetic duplexes and the genomic SSRs was the number of RUs.

## Results and Discussion

### Minimization of artifacts in CCS spanning an SSR within a synthetic duplex

**Figure 1:**
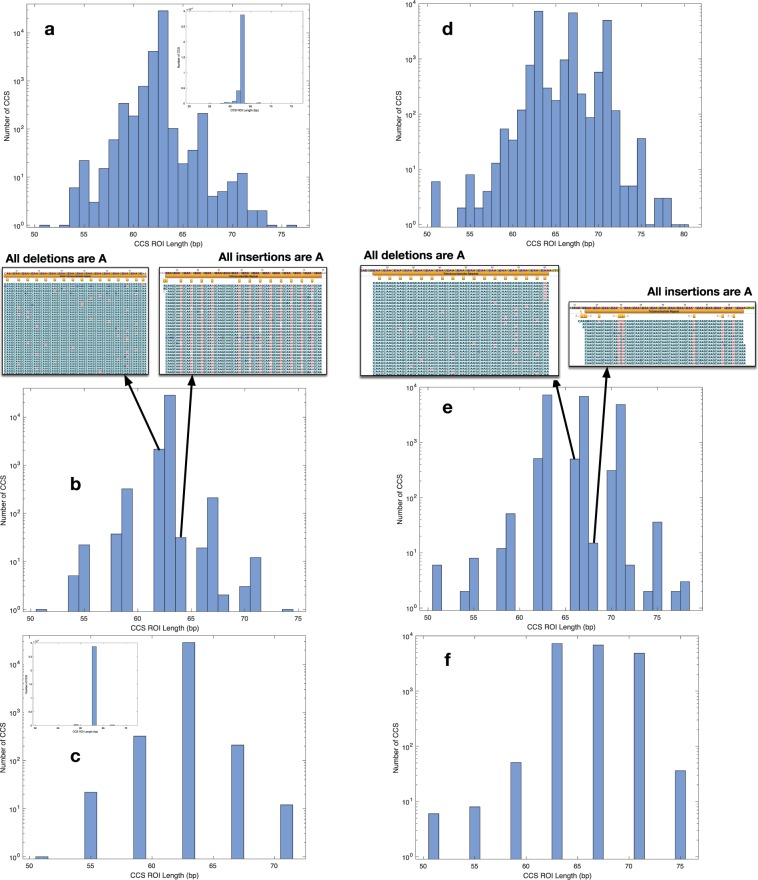
Histograms showing the effect of the fraction base composition (FBC) filter at various z-score settings, on CCS mapping to the AAGC SSR in control duplexes to assess the effects of SSM in CCS sequencing. Only CCS passing initial filters for length and quality are shown. Panels a–c show histograms of CCS from a single 63 bp (16 RU-1 bp) synthetic control SSR. (**a**) No z-score filter applied (inset is same histogram with linear ordinate). (**b**) Z-score = 2.5, CCS in bins at non-integer RU distances from the control SSR length consists of adenine deletions or insertions. (**c**) Z-score = 1.5 showing a dominant mode of 63 bp corresponding to control SSR length and minor modes at integer RU distances due to SSM during sequencing (inset is same histogram with linear ordinate). Panels d–f show histograms of CCS from a mixture of libraries of synthetic duplexes with 63, 67, and 71 bp length SSRs. d. No z-score filter applied. (**e**) Z-score = 2.25 with CCS comprising single base indels relative to CCS at the control SSR lengths or in sidebands of bins at integer number of RUs different from the control SSR lengths. (**f**) Z-score = 1.5 showing occupancy of control SSR length modes at 63, 67, 71 bp and minor modes at 51, 55, 59, 75 bp due to SSM of the sequencing polymerase.

In Fig. 1, we show histograms of the number of CCS observed at multiple mapped SSR lengths, both before and after filtering the data (see Methods). All data, including the tabular data (.csv files) used to generate the histograms, are available in the relevant directories in the Reproducibility pane within the CodeOcean compute capsule^16^ (see README.md in compute capsule and Methods). First, a library consisting of a single 63 base (16 RU-1 bp, 63 bp) SSR (Fig. 1a–c) was sequenced and the CCS were mapped to the control sequence to derive a set of CCS ROI. These CCS ROI were quality filtered (see Methods) with the number of the observed mapping CCS plotted on a logarithmic scale (see inset for linear scale) vs the length of the mapping CCS ROI. In Fig. 1a, no FBC z-score filter is applied which presents a single dominant mode at the expected length (made prominent by using a linear ordinate in the inset histogram). Also evident are minor numbers of insertions or deletions of 1–3 bases that are the main error mode of the CCS protocol, with deletions more common.

Applying an FBC z-score threshold of 2.5 to the same data (Fig. 1b) removes some CCS but retains 99.53% of the unfiltered CCS in the 63 bp control SSR length bin. Remaining CCS in the bins 1 bp less or more than the control SSR length (we define as 1 bp sidebands) consisted entirely of adenine deletions or insertion (arrow call-outs in Fig. 1b). The sidebands for the bins observed for modes 1 RU larger or smaller than the control SSR length mode are also the result of adenine insertion/deletions, indicating this is the primary error mode for CCS sequencing in this AAGC repeat context. In Fig. 1c, the FBC z-score threshold of 1.5 eliminated the sidebands due to CCS error and highlighted the primary control SSR length mode and CCS in bins at +/−1, 2 and −3 RU. Because the input DNA fragments were a homogeneous, synthetic population with a 63 bp SSR, the other CCS in +/−1, 2 and −3 RU bins were necessarily due to SSM during sequencing, and occurred with a frequency relative to the control SSR length mode of 1.13% at −1 RU (59 bp), 0.736% at +1 RU (67 bp), 0.077% at −2 RU (55 bp), 0.042% at +2 RU (71 bp). The FBC z-score threshold retained 95% of the control SSR length CCS after both filters were applied. Thus, the FBC z-score filter is a mechanism that reduces the inherent artifacts of the sequencing process so that variation in the apparent number of synthetic duplex DNA molecules with SSR RU number can be estimated.

### Minimization of artifacts in CCS spanning individual SSRs from a mixture of three synthetic duplexes

The suitability of this FBC-based filter for mixtures of SSR length was examined to demonstrate the minimization of sequencing artifacts between multiple SSR lengths from a single DNA sample. Three duplex libraries containing 15, 16, or 17 RU were pooled in a 2:4:1 mole ratio, respectively, before library preparation and CCS sequencing. The diffusion loading of the ZMWs is non-linear with respect to library size and concentration and we note that our < 100 bp duplexes inserts are a non-canonical use of the technology that is currently being used to sequence inserts >5 kb. This loading behavior does not affect the SSR results acquired from the *H. somni* CCS genomic inserts in this study (average length = 2467 bp, standard deviation = 1061.4 bp, 841 X coverage) where each CCS was required to span the entire extent of the SSRs and SSM variants if present. The data were analyzed in the same fashion as for the single duplex (Fig. 1d–f) and demonstrated the same characteristics with respect to sidebands and adenine insertion/deletion (Fig. 1e), and artifact SSM (Fig. 1f), occurring with similar frequencies. The impact of FBC z-score on histogram distribution was explored (see the CodeOcean compute capsule)^16^ and demonstrates that once the 1 bp sideband modes are removed with a FBC z-score of around 1.5, the histograms do not change until the FBC z-score is set to below 1. The breadth of 0.5 (1.5 to 1) in the FBC z-scores yielding identical CCS length distributions demonstrates that the filter should provide a reasonably straightforward path to remove artifact and that this method is not excessively dependent on the precise choice of FBC z-score. Practically speaking, the FBC z-score is adjusted from large to small until all of the side bands are removed. These data suggest that the FBC z-score filter provides a robust estimate of the actual length heterogeneity in a population of genomic SSRs using CCS sequencing, by controlling for the occurrence of sequencing process-induced artifacts.

### Minimization of artifacts in CCS spanning SSRs within genomic DNA reveals SSR phase and length variants

**Figure 2:**
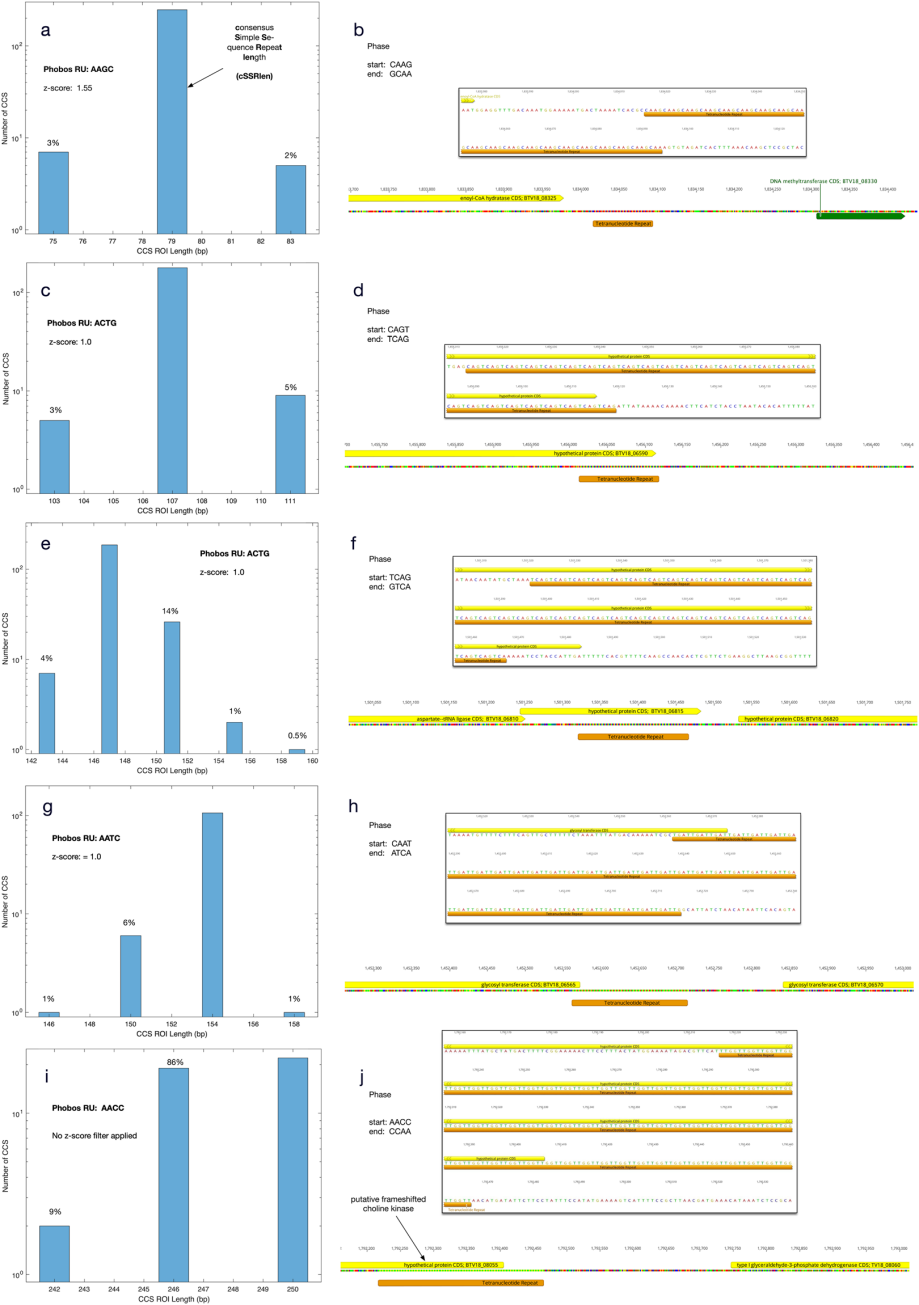
Histograms and sequence contexts for tetranucleotide SSRs, where SSR sequence phase is defined by the first and last tetramers in the SSR. The percentage of CCS in non-consensus SSR length (cSSRlen) modes relative to those in the dominant cSSRlen modes are designated above their respective bins. (**a**) The CCS mapping to the 79 bp AAGC SSR using an FBC z-score = 1.55. (**b**) The genome context of the 79 bp AAGC SSR. (**c**,**d**) Same as the preceding panel pair but for the 107 bp ACTG SSR with FBC z-score = 1.0. (**e**,**f**) As with the preceding panel pairs, but for the 147 bp ACTG SSR. (**g**,**h**) As with preceding panel pairs, but for the 154 bp AATC SSR. (**i,j**) As with the preceding panel pairs, but for the 250 bp AACC SSR with no FBC filter applied.

The success of calibrating the error motivated us to evaluate SSR length variation in the more complex case of the parent *H. somni* bacterial genome. The genome assembly (accession CP018802) contains five tetranucleotide repeats >10 RU in length (ranging from 107–250 bp), including the SSR represented by the synthetic constructs described above. The two-step filter was applied to CCS that mapped to these SSRs, adjusting the FBC z-score independently for each of the five repeat regions (Fig. 2). The histograms of all five SSR have dominant modes at the consensus SSR length (cSSRlen) depicted in the genome assembly, consistent with the consensus assembly representing the most common number of RU. SSR lengths representing integer changes of RU number, consistent with an SSM model of SSR length variation^17^, are evident after applying the filters to reduce sequencing process-induced artifact and indicate that SSR lengths are best represented by a probability metric rather than a static single length measure. Occupancy of all but one of the non-cSSRlen modes (Fig. 2g, 158 bp, cSSRlen +1 RU) occurred at a frequency greater than the predicted rate of sequence process-induced SSM in the control 63 bp SSR. While it is likely that sequencing process-induced SSM occurs at a greater frequency as the SSR length increases, the CCS of genomic DNA still strongly support the existence in the culture of individual cells with variable SSR length profiles.

We observed tetranucleotide SSR RU numbers ranging from +3 RU longer than cSSRlen (Fig. 2e) to −2 RU shorter (Fig. 2i), with substantial variation in the relative frequencies of the expanded or contracted subpopulations between different genomic locations. We note that the 79 (Fig. 2a) and 107 bp (Fig. 2b) SSRs each show 2 subpopulations of CCS at +/−1 RU, while the 147 (Fig. 2e) and 154 bp (Fig. 2g) SSRs show 4 and 3 distinct subpopulations of CCS respectively. This observation is consistent with the hypothesis that increased SSR length provides increased opportunity for SSM^18^. The 250 bp SSR (Fig. 2i) does not follow the trend with further increases of distinct subpopulations at this SSR, but rather CCS were more evenly redistributed between the −1 RU mode (86% of cSSRlen) and cSSRlen mode, while the CCS at −2 RU constitute 9% of the cSSRlen mode. We conclude that SSM can affect the breadth of the SSR length populations (e.g. Fig. 2a,b vs 2e), the skew of SSR length populations (e.g. Fig. 2e vs 2g), and the relative occupancy of different subpopulations (e.g. Fig. 2i vs 2a,c,e,g). The effect of strain or environmental conditions (e.g. culture medium) on these characteristics may provide further insight into the role of genome plasticity on adapability. The effect of the sequence context on SSM cannot be determined from the single isolate examined, but we note that the 250 bp SSR is of low complexity (containing only A and C) which we speculate may be related to the increased SSM rate observed.

The positions of the five tetranucleotide repeats relative to annotated features of the genome assembly (Fig. 2 panels b,d,f,h,j) identify four that overlap open reading frames (ORF). One overlaps the carboxy-terminal end of a glycosyl transferase coding region, the other three overlap hypothetical protein ORF. The potential biological impact of SSR variation was examined by analysis of the 250-bp cSSRlen repeat (Fig. 2i) and the overlapping ORF encoding hypothetical protein BTV18_08055 (Fig. 2j). The hypothetical protein has predicted function as a choline kinase according to BLASTX analysis against the RefSeq Proteins database. Changes in the RU number of this SSR can introduce frame shifts in the protein coded by the predicted transcript, potentially affecting function. Choline kinases have been reported to be an important virulence factor in *H. somni* and other species^19,20^ thus, the modulation of SSR length could conceivably impact virulence.

**Figure 3:**
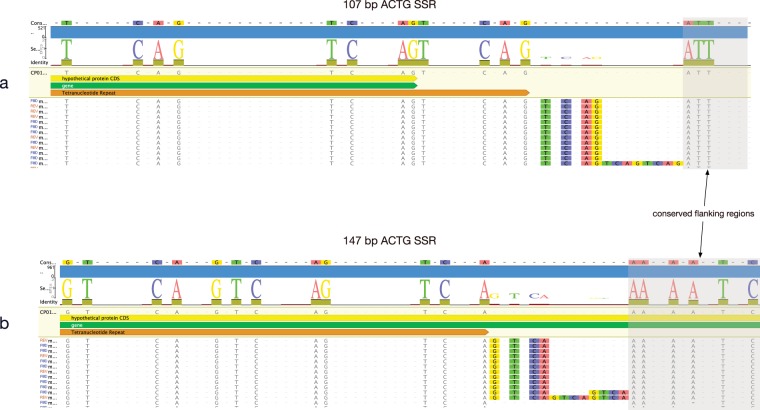
Comparison of the 3′-ends of CCS mapping to two SSRs comprised of ACTG RUs. (**a**) The 107 bp SSR terminates with CCS ending with TCAG and is initiated with a CAGT upstream starting sequence (Fig. 2d). (**b**) In contrast, the 147 bp SSR terminates with CCS ending with GTCA and is initiated with a TCAG (Fig. 2f).

Two of the *H. somni* SSR are comprised of the same RU sequence (ACGT; Fig. 2c,e), yet have substantially different SSM-mediated SSR length subpopulation frequency profiles. This could be a direct consequence of total repeat length, but we considered the possibility that it is a consequence of repeat phase (Fig. 2d,f). The SSR with 107 bp cSSRlen has phase CAGT if defined at the 5′-end, but TCAG if defined by the 3′-end. In contrast, the SSR with 151 bp cSSRlen has phase TCAG defined at the 5′-end, but GTCA if defined by the 3′-end (Fig. 2d,f). Closer examination of the 3′-ends of these two SSRs confirms variation of CCS populations ending with TCAG (Fig. 3a) or GTCA (Fig. 3b). There is insufficient evidence to definitively attribute the variation in the SSR length subpopulation distribution profiles to sequence phase difference. However, we believe this is the first demonstration of SSR sequence phase differences in association with SSM-mediated SSR length frequency distribution profiles in any organism and suggest that the phenomenon may play a role in the biology of microbial genomes.

## Conclusions

The methods presented demonstrate that sequencer systematic error can be estimated to yield quantitative metrics associated with the effects of SSM on tetranucleotide repeat length distributions. This approach may yield similar results for other SSRs with different RU sequences and lengths. This PCR-free approach was developed to characterize changes in a bacterial isolate’s SSR length distribution(s) when subjected to an environmental perturbation, where the effects of SSR RU sequence, RU length, and genomic context will be identical between conditions, so that the effect of the perturbation on a given locus’s SSR length distribution can be evaluated. We note that the FBC filter requires that the RU be comprised of at least 2 different bases to be effective, so a mononucleotide SSR would not be an appropriate target. The relationships between these metrics and adaptability or other biology require dedicated experiments utilizing these methods with the goal of understanding the effect of culture condition and genomic components on the observables demonstrated here. These results suggest that SSM-mediated phase variation in bacterial populations inherently contain quantifiable subpopulations, each with their own environmental fitness, whose fittest members can be tracked as they rapidly expand upon environmental perturbation providing new insights into adaptability biology. The use of control DNA duplexes is appropriate not only for single-molecule sequencing using SMRT sequencing but could be gainfully employed to assess sequencing artifact using other next-generation sequencing technologies.

## Methods

### *Histophilus somni* USDA-ARS-USMARC-63250 culture and DNA isolation

*H. somni* isolates were previously sequenced using SMRT sequencing as previously described^21^ for consensus genome assembly and identification of SSRs. For the present study, the USDA-ARS-USMARC-63250 strain was revived from a −80 °C glycerol stock by streaking onto Chocolate II agar plates (Becton Dickinson Co. Sparks, MD) in 5% CO_2_ at 37 C for 24–48 h. An individual, well-separated colony was used to inoculate a 10 ml culture of brain heart infusion (BHI) broth supplemented with 2X Veterinary Fastidious Medium (Thermo Fisher Scientific, Waltham, MA)(Thermo Scientific, Waltham, MA) in a 50 ml conical tube, which was incubated at 37 °C with shaking at ~190 rpm for 24 h. Cells were pelleted and genomic DNA extracted using the Qiagen DNeasy Blood & Tissue kit and Genomic-tip 100/G columns according to the manufacturer’s protocol. Genomic DNA quantity and quality were assessed by spectrophotometry using a NanoDrop (Thermo Fisher, Waltham, MA).

### CCS sequencing

Sequencing libraries were prepared with SMRTbell Template Prep Kit 1.0 as directed by the manufacturer (Pacific Biosciences, Menlo Park, CA). Briefly, sheared and end-repaired genomic DNA fragments, or annealed synthetic SSR oligonucleotides, were ligated to SMRTbell adapter sequences, purified from the ligation mix, and quantified by fluorescence using a DS-11 FX + fluorometer (Denovix, Wilmington, DE). No amplification steps were performed. The libraries of standard duplex were diffusion loaded, while the bacterial genomic DNA libraries were MagBead loaded. Sequencing (CCS) was performed on an RSII instrument using P6/C4 chemistry (Pacific Biosciences, Menlo Park, CA) with base calling and processing of individual ZMW data to CCS performed within SMRT analysis v2.3.0.

### Control duplex synthesis

The three control duplexes were based on the 79 bp AAGC SSR found in strain USDA-ARS-USMARC-63250 (GenBank accession CP018802) between positions 1,834,016–1,834,094 as determined by the Phobos tandem repeat finder^22,23^ using normalized alphabetical mode. The control duplexes were annealed from chemically synthesized oligonucleotide sequences. The duplexes were ordered to be delivered annealed by the manufacturer (Integrated DNA Technologies, Coralville, IA) and are presented in 5′->3′ orientation below. Graphical depictions of the base-paired duplexes are also presented at our protocols.io site^24^.

**NM2 duplex**

seq. 1: GAC TAA AAT CAC GCC AAG CAA GCA AGC AAG CAA GCA AGC AAG CAA GCA AGC AAG CAA GCA AGC AAG CAA GCA AGC AAG CAA GCA AAG TGT AGA TCA

seq. 2: AAG TGA TCT ACA CTT TGC TTG CTT GCT TGC TTG CTT GCT TGC TTG CTT GCT TGC TTG CTT GCT TGC TTG CTT GCT TGC TTG CTT GGC GTG ATT TTA

**NM3 duplex**

seq. 1: GAC TAA AAT CAC GCC AAG CAA GCA AGC AAG CAA GCA AGC AAG CAA GCA AGC AAG CAA GCA AGC AAG CAA GCA AGC AAG CAA AGT GTA GAT CA

seq. 2: AAG TGA TCT ACA CTT TGC TTG CTT GCT TGC TTG CTT GCT TGC TTG CTT GCT TGC TTG CTT GCT TGC TTG CTT GCT TGC TTG GCG TGA TTT TA

**NM4 duplex**

seq. 1: GAC TAA AAT CAC GCC AAG CAA GCA AGC AAG CAA GCA AGC AAG CAA GCA AGC AAG CAA GCA AGC AAG CAA GCA AGC AAA GTG TAG ATC A

seq. 2: AAG TGA TCT ACA CTT TGC TTG CTT GCT TGC TTG CTT GCT TGC TTG CTT GCT TGC TTG CTT GCT TGC TTG CTT GCT TGG CGT GAT TTT A

### CCS mapping and processing to create FASTQ of CCS ROI

A flowchart depicting the overall computational scheme, the precise steps for processing the control CCS, as well as input and output data are available at Steps to Create FASTQ of CCS Overlapping Control SSR-CCS ROI (protocols.io)^24^. The control CCS were mapped using Geneious (v 11.1.5) Assembler^25^ to their source DNA sequence with the resulting alignment processed with bedtools intersect^26,27^ to identify CCS completely spanning the SSR and 5 bp flanking region. Geneious was subsequently used to trim flanking non-SSR regions and create a FASTQ file of CCS spanning only the SSR, defined to be the region of interest (ROI). Nine CCS libraries from the *H. somni* USDA-ARS-USMARC-63250 strain (GenBank accession CP018802) were sequenced to 841x coverage and processed into a BAM file as described at BAM Alignment to CP018802 for Submission to GenBank^28^ and is available under accession SRR8080935. The CCS from this BAM file was mapped to the genome and processed in an analogous manner as the control CCS with the data, flowcharts, and detailed protocol provided at Steps to Create FASTQ of CCS Overlapping Genomic SSR-CCS ROI^29^.

### Computing quality and fraction base content filtered CCS and identification of the slipped strand mispairing artifact

The FASTQ files created in the previous mapping and trimming step were processed using scripts in the MATLAB computing environment. The input data was processed by the MATLAB scripts in this CodeOcean compute capsule^16^. All of the scripts, their input and output data, including tabulated data supporting the histograms presented, are available for download in this compute capsule. Within the capsule’s README.md file are flowcharts summarizing the processes and instructions for running the compute capsule. See the README.md within the CodeOcean compute capsule for more information about using and navigating this resource. A quality filter was used to remove CCS if any base had a quality score of under 20 or an average base quality score under 30. The fraction base composition vectors were computed with elements defined according to:

$$FB{C}_{i=1}^{n(CCS)}(B=A,C,G,T)=\frac{n({B}_{i})}{n({A}_{i})+n({C}_{i})+n({G}_{i})+n({T}_{i})}$$

where *n*(*B*_*i*_) is the number of occurrences of base *B* in *CCS i* and *n*(*CCS*) is the number of CCS with acceptable quality spanning the SSR. Once the vector elements were computed, the absolute value of the FBC z-score threshold was iteratively adjusted, usually from larger to smaller values, to identify and remove CCS exceeding the FBC threshold for each base.

## Data Availability

All data, code, and computed results are available at the protocol.io^24,29^ and Code Ocean compute capsule^16^ links provided in the Methods section.
