# Publisher Correction: A Computational Method to Quantify the Effects of Slipped Strand Mispairing on Bacterial Tetranucleotide Repeats

**DOI:** 10.1038/s41598-020-58622-2

**Published:** 2020-01-28

**Authors:** Gregory P. Harhay, Dayna M. Harhay, James L. Bono, Sarah F. Capik, Keith D. DeDonder, Michael D. Apley, Brian V. Lubbers, Bradley J. White, Robert L. Larson, Timothy P. L. Smith

**Affiliations:** 1 USDA ARS US Meat Animal Research Center, Clay Center, NE United States; 2 Texas A&M AgriLife Research, Amarillo, TX and the College of Veterinary Medicine & Biomedical Sciences, Texas A&M University System, College Station, TX United States; 3 Veterinary and Biomedical Research Center, Inc, Manhattan, KS United States; 4 Kansas State University, College of Veterinary Medicine, Manhattan, KS United States

Correction to: *Scientific Reports* 10.1038/s41598-019-53866-z, published online 02 December 2019

This Article contains a typographical error in the Introduction section.

“Where this postulate holds true, the fractional base compositions (FBC, the proportion of the number of A, C, G, or T relative to the total number of bases in the SSR) for a base in SSM-mediated SSR length variants are necessarily nearly identical and essentially independent of the total length of the SSR, given the SSR > RU length.”

should read:

“Where this postulate holds true, the fractional base compositions (FBC, the proportion of the number of A, C, G, or T relative to the total number of bases in the SSR) for a base in SSM-mediated SSR length variants are necessarily nearly identical and essentially independent of the total length of the SSR, given the SSR > > RU length.”

